# Response to: “DOACs for Left Ventricular Thrombus: Persistent Equipoise Despite New Observational and Randomized Data”

**DOI:** 10.1016/j.rpth.2025.103340

**Published:** 2025-12-29

**Authors:** Hossam Elbenawi, Kirsten Lipps, Samuel Heller, David A. Liedl, Raymond C. Shields, Ana I. Casanegra, Stanislav Henkin, Thom W. Rooke, Paul W. Wennberg, Waldemar E. Wysokinski, Robert D. McBane, Damon E. Houghton

**Affiliations:** 1 Division of Vascular Medicine, Department of Cardiovascular Medicine, Mayo Clinic, Rochester, Minnesota, USA; 2 Gonda Vascular Center, Mayo Clinic, Rochester, Minnesota, USA; 3 Second Faculty of Medicine, Charles University, Prague, Czech Republic; 4 Division of Hematology, Department of Internal Medicine, Mayo Clinic, Rochester, Minnesota, USA

We commend Dziewierz et al. [1] for their insightful Letter to the Editor summarizing the latest evidence on the comparative effectiveness and safety of direct oral anticoagulants (DOACs) vs vitamin K antagonists (VKAs) in patients with left ventricular thrombus (LVT). They highlight the ongoing uncertainty surrounding DOAC use, despite emerging observational and randomized clinical trials that address key clinical outcomes, such as systemic embolic events (SSEs) and major bleeding.

We agree on the need for prospective, larger studies to help compare different types of anticoagulants in patients with LVT, and we acknowledge that the retrospective design of our study limits the strength of our conclusions. Until definitive prospective studies can be conducted, we believe that our manuscript [2] addresses methodological concerns raised by other retrospective studies and offers new insights not considered in existing prospective studies. Unlike other retrospective studies, patients who received more than 1 type of oral anticoagulant during follow-up (ie, switched between DOACs and VKAs) were excluded from our study, as were those already on chronic anticoagulation at the time of LVT diagnosis. This approach ensured that the observed outcomes could be attributed to a single type of oral anticoagulant, and all patients were newly initiated on anticoagulation for this diagnosis.

Dziewierz et al. [1] highlight, for comparison, the Retrospective Evaluation of DOACs and Vascular Endpoints of Left Ventricular Thrombi (RED VELVT) study [3], which reported a higher SSE risk among patients treated with DOACs, with rates of 9.1% for DOACs and 4.6% for VKAs. Our findings [2], along with those of others [[4], [5], [6], [7]], indicate that DOACs were associated with a risk of SSEs similar to that of VKAs (DOAC 5.7% vs VKA 5.4%; *P* = 1.00). This discrepancy may reflect differences in study design and patient populations, but it may also indicate biased outcome assessment and immortal time bias in the RED VELVT study. In that study [3], SSEs that occurred either initially or during follow-up with bridging or parental anticoagulation were not attributed to the warfarin group. Since bridging with parental anticoagulants is rare with DOACs but a mainstay of warfarin management, events occurring during the high-risk period of initiation or temporary anticoagulation interruption would be attributed to DOAC therapy rather than to warfarin therapy in most cases. Additionally, immortal time bias may be introduced at the onset of anticoagulation treatment with warfarin, since it may take 5 to 10 days of initial bridging therapy, where events and deaths would not be attributed to warfarin. Our study design eliminates these biases.

The higher prevalence of atrial fibrillation and prior SSEs in RED VELVT could account for the overall increased rate of SSEs, especially later events. Furthermore, patients who transitioned between DOAC and VKA therapies were included in the analysis and classified according to the agent used at the time of the event, potentially influencing estimates. In contrast, we attributed all SSEs following initiation of either DOAC or VKA therapy to the assigned oral anticoagulant, even when bridging was used during VKA initiation (of 6 SSEs associated with VKAs, 2 occurred during bridging with intravenous heparin). We included only patients who remained on a consistent anticoagulation regimen throughout follow-up, excluding those who switched between DOACs and VKAs. This stricter methodological approach may account for some of the observed differences in outcomes between the studies.

Dziewierz et al. [1] also contrast our study results with the findings of the RIVAWAR randomized, open-label trial [8], noting numerically higher (though not statistically significant) rates of both ischemic stroke (3.5% vs 1.1%; *P* =.43) and major bleeding (2.3% vs 1.1%; *P* =.66) with rivaroxaban 20 mg daily compared with warfarin, with a target international normalized ratio of 2 to 3. Recent letters from Abdelnabi et al. [9] and Shah et al. [10] further debate the merits of the RIVAWAR study. In our study, among 70 patients treated with DOACs, most received apixaban (91.4%) rather than rivaroxaban. The RIVAWAR trial reported that all 171 patients were treated with rivaroxaban 20 mg once daily in addition to dual antiplatelet therapy. Our study results, although not definitive, examined the potential implications of initiating atrial fibrillation-based initial dosing vs acute thrombosis dosing regimens (ie, “loading” doses). The higher rate of major bleeding in our study compared with the RIVAWAR trial may be explained by several factors. First, our study included a much longer follow-up period, whereas RIVAWAR reported outcomes only through 12 weeks, providing fewer opportunities to capture bleeding events. Second, RIVAWAR excluded patients with a high bleeding risk (prior major or intracranial bleeding, cardiogenic shock at the time of LVT, and advanced chronic kidney disease [patients with glomerular filtration < 30 mL/min]), whereas we applied broader inclusion criteria, likely resulting in the enrollment of patients with greater baseline bleeding susceptibility. Finally, the predominant use of apixaban in our cohort, compared with rivaroxaban in RIVAWAR, introduces differences in drug-specific bleeding profiles that may also account for the observed discrepancy.

Unfortunately, in addition to methodological differences, the smaller sample sizes of both retrospective and prospective studies limit the precision of bleeding and stroke/systemic embolization rates in patients with LVT. Powering future studies based on the outcome of LVT resolution is inadvisable, as it is ultimately a surrogate outcome that may not address meaningful patient outcomes. The expected difference in SSEs between DOACs and VKAs, based on existing data, suggests that any absolute differences in outcomes are likely small, indicating that large, well-funded studies would be required to prove a difference (or noninferiority) in outcomes, such as SSEs. We are not aware of any active or planned randomized controlled trials to definitely answer this question. Prior meta-analyses [11,12] have attempted to overcome the challenges posed by smaller studies, but with numerous new publications since then, a more comprehensive and detailed systematic review and meta-analysis to pool all the evidence is urgently needed.

## References

[1] Dziewierz A., Jarosz P., Rakowski P. (2025). DOACs for left ventricular thrombus: persistent equipoise despite new observational and randomized data. Res Pract Thromb Haemost.

[2] Elbenawi H., Lipps K., Heller S., Liedl D.A., Shields R.C., Casanegra A.I. (2025). Comparison of direct oral anticoagulants and vitamin K antagonists in the treatment of left ventricular thrombi, a retrospective cohort study. Res Pract Thromb Haemost.

[3] Robinson A.A., Trankle C.R., Eubanks G., Schumann C., Thompson P., Wallace R.L. (2020). Off-label use of direct oral anticoagulants compared with warfarin for left ventricular thrombi. JAMA Cardiol.

[4] Abdelnabi M., Saleh Y., Fareed A., Nossikof A., Wang L., Morsi M. (2021). Comparative study of oral anticoagulation in left ventricular thrombi (No-LVT Trial). J Am Coll Cardiol.

[5] Seiler T., Vasiliauskaite E., Grüter D., Young M., Attinger-Toller A., Madanchi M. (2023). Direct oral anticoagulants versus vitamin K antagonists for the treatment of left ventricular thrombi-insights from a Swiss multicenter registry. Am J Cardiol.

[6] Jones D.A., Wright P., Alizadeh M.A., Fhadil S., Rathod K.S., Guttmann O. (2021). The use of novel oral anticoagulants (NOAC) compared to vitamin K antagonists (warfarin) in patients with left ventricular thrombus after acute myocardial infarction (AMI). Eur Heart J Cardiovasc Pharmacother.

[7] Willeford A., Zhu W., Stevens C., Thomas I.C. (2021). Direct oral anticoagulants versus warfarin in the treatment of left ventricular thrombus. Ann Pharmacother.

[8] Shah J.A., Hussain J., Ahmed B., Batra M.K., Ali G., Naz M. (2025). Rivaroxaban vs warfarin in acute left ventricular thrombus following myocardial infarction: RIVAWAR, an open-label RCT. JACC Adv.

[9] Abdelnabi M., Almaghraby A., Ibrahim R., Ayoub C., Arsanjani R. (2025). RIVAWAR trial commentary: Rivaroxaban vs warfarin in acute left ventricular thrombus following myocardial infarction. JACC Adv.

[10] Shah J.A., Karim M., Hakeem A., RIVAWAR investigators (2025). Reply: RIVAWAR trial commentary: Rivaroxaban vs warfarin in acute left ventricular thrombus following myocardial infarction. JACC Adv.

[11] Hu T., Chen C., Maduray K., Han W., Chen T., Zhong J. (2024). Comparative effectiveness and safety of DOACs vs. VKAs in treatment of left ventricular thrombus- a meta-analysis update. Thromb J.

[12] Kitano T., Nabeshima Y., Kataoka M., Takeuchi M. (2023). Trial sequential analysis of efficacy and safety of direct oral anticoagulants and vitamin K antagonists against left ventricular thrombus. Sci Rep.

